# Passive ventricular remodeling in cardiac disease: focus on heterogeneity

**DOI:** 10.3389/fphys.2014.00482

**Published:** 2014-12-22

**Authors:** Elise L. Kessler, Mohamed Boulaksil, Harold V. M. van Rijen, Marc A. Vos, Toon A. B. van Veen

**Affiliations:** ^1^Division of Heart and Lungs, Department of Medical Physiology, University Medical Center UtrechtUtrecht, Netherlands; ^2^Department of Cardiology, Radboud University Medical CenterNijmegen, Netherlands

**Keywords:** gap junction, sodium channel, fibrosis, arrhythmias, heterogeneity

## Abstract

Passive ventricular remodeling is defined by the process of molecular ventricular adaptation to different forms of cardiac pathophysiology. It includes changes in tissue architecture, such as hypertrophy, fiber disarray, alterations in cell size and fibrosis. Besides that, it also includes molecular remodeling of gap junctions, especially those composed by Connexin43 proteins (Cx43) in the ventricles that affect cell-to-cell propagation of the electrical impulse, and changes in the sodium channels that modify excitability. All those alterations appear mainly in a heterogeneous manner, creating irregular and inhomogeneous electrical and mechanical coupling throughout the heart. This can predispose to reentry arrhythmias and adds to a further deterioration into heart failure. In this review, passive ventricular remodeling is described in Hypertrophic Cardiomyopathy (HCM), Dilated Cardiomyopathy (DCM), Ischemic Cardiomyopathy (ICM), and Arrhythmogenic Cardiomyopathy (ACM), with a main focus on the heterogeneity of those alterations mentioned above.

## Introduction

The lifelong purpose of the heart is maintenance of cardiac output and supply of all organs with an appropriate amount of oxygen and nutrients. At the same time it steers the controlled homeostasis of the blood via clearance of waste products, drugs and imbalanced levels of ions and proteins in the lungs, liver, and kidneys. To be able to fulfill this task, the heart has to adapt its workload to the needs of the body (Kemp and Conte, 2012). Many different diseases like coronary artery disease, myocardial infarction, hypertension, dysfunction of valves, congenital heart disease, lung diseases, diabetes, anemia, hyperthyroidism, and or arrhythmia/dysrhythmia can alter the performance of the heart (Lloyd-Jones et al., 2002; van Rijen et al., 2006; Roger et al., 2011). In order to preserve cardiac output under these conditions, the heart will start several compensatory mechanisms, like the Frank Starling mechanism, neurohumoral activation, and inflammational responses (Westerhof and O'Rourke, 1995; Lee and Tkacs, 2008). Moreover, the heart starts to modify the gene program that shapes the individual cells that compose the organ. This in an attempt to adapt chronically to the new requirements of its environment. These adaptations together will result in myocardial remodeling that most often has heterogeneous characteristics (Swynghedauw, 1999).

By nature, the heart itself is a rather heterogeneous organ: morphologically, the right and left side as well as atria and ventricles show various differences in cell constellations, hemodynamics and their respective electrical characteristics (Zimmer, 1994). The different resistances in peripheral and pulmonary circulation for instance are causative for the differences between left and right ventricle anatomy (right ventricular wall is much thinner than the left wall). Also with respect to molecular make-up and microarchitecture of the cardiac muscle significant differences exist. The way the fibers are orientated within the left and right ventricle is not comparable. Electrical impulse propagation faces differences in conduction velocity between left and right ventricles but also with the specialized conduction system. Part of that relies on differences in expression level and pattern of gap junction channels and the ion channels that underlie action potential generation.

The sodium channel protein Nav1.5, for instance, is heterogeneously distributed throughout the ventricles and the cardiac conduction system in healthy hearts (Remme et al., 2009). Besides that, two pools of Nav1.5 channels can be found in cardiomyocytes: a lateral pool and a pool in the intercalated disc that co-exist and interact with different proteins (Petitprez et al., 2011). Also Cx43 is found in different densities within the healthy heart e.g., regarding the posterior and anterior wall of the ventricles (Strom et al., 2010).

Beyond the natural heterogeneity that is found under the normal physiological functioning of the heart, this review will focus on the heterogenic aspect that is involved in passive remodeling of the ventricles under pathophysiological conditions. Passive remodeling is defined as the chronic molecular and structural adaptations in ventricular cardiomyocytes and alterations in gene expression as induced by different forms of heart disease. Active remodeling, in contrast, is defined as phosphorylation processes that e.g., result from sympathic and parasympathic imbalance. This is exampled by the increase in heart rate that is facilitated through alterations in phosphorylation of ion channels (e.g., L–type calcium channels) in the sinus node.

One of the most crucial and maladaptive factors of passive remodeling in the ventricles is heterogeneity in electrical and structural remodeling. In that perspective we will discuss factors associated with conduction and propagation of excitation, but not with respect to repolarization (e.g., potassium currents, heterogeneity in action potential duration), and contraction (e.g., calcium-handling and sarcomeric proteins). During remodeling, heterogeneous alterations in three factors contribute to increase the propensity to arrhythmias and to develop heart failure: (1) tissue architecture such as hypertrophy, fibrosis, fiber disarray, and cell size, (2) electrical coupling by means of gap junctions and especially those composed of Cx43, and (3) electrical excitability due to changes in sodium channels that are mainly composed of Nav1.5 (Kleber and Rudy, 2004; van Rijen et al., 2006; Bowers et al., 2010).

We will address remodeling of these factors during Dilated Cardiomyopathy (DCM), Hypertrophic Cardiomyopathy (HCM), Ischemic Cardiomyopathy (ICM) and Arrhythmogenic Cardiomyopathy (ACM), previously known as Arrhythmogenic Right Ventricular Cardiomyopathy/Dysplasia (ARVC/D). In general, DCM can be identified by cardiac chamber dilatation and reduced systolic function often leading to congestive heart failure. It is the most commonly occurring cardiomyopathy in adults and children and is associated with muscle dysfunction and/or volume overload. On the other hand, in children DCM is mostly caused by myocarditis and neurohumoral diseases (Towbin et al., 2006). DCM is defined as the presence of left ventricular fractional shortening (<25%) and/or LVEF < 45% and left ventricular end-diastolic dimensions (LVEDD) of >117% of the predicted value by the Henry formula (Mestroni et al., 1999). It can also be caused by a variety of genetic mutations that are uncovered upon analysis of family history and molecular genetic testing. These forms, however, are referred to as familial dilated cardiomyopathy (FDC) (Hershberger and Morales, 2013). HCM has been defined by the World Health Organization as the presence of left or biventricular hypertrophy, in absence of any cardiac or systemic cause (Richardson et al., 1996). When this definition is used, the general prevalence is about 1:500 (Maron, 2002). However, HCM can also be caused by mutations in genes encoding for cardiac sarcomeric and myofilament proteins; for the latter already more than 1400 mutations have been identified increasing the prevalence even more (Maron et al., 2012; Efthimiadis et al., 2014).

ICM results from myocardial ischemia and is characterized by remodeling due to myocardial infarction which eventually triggers loss of contractility, still being the leading cause of ventricular dysfunction worldwide (reviewed in Wu, 2007).

Finally, ACM is a non-ischemic progressive and predominantly heritable heart disease associated with cardiac arrhythmias and sudden cardiac death (Corrado et al., 2000). ACM is characterized through replacement of cardiomyocytes by fibro-fatty tissue (Thiene et al., 1988; Saffitz et al., 2009). About 60% of the cases have a hereditary basis and mutations causing ACM have been found in several desmosomal genes like desmoplakin (Rampazzo et al., 2002), plakoglobin (Asimaki et al., 2007), plakophilin2 (Gerull et al., 2004; van Tintelen et al., 2006), desmocollin2 (Heuser et al., 2006; Syrris et al., 2006), and desmoglein (Pilichou et al., 2006). Moreover, genes not related to the desmosome can be affected such as the transmembrane protein 43 (TMEM43), phospholamban (PLN), desmin and transforming growth factor beta3 (TGFβ3) (Beffagna et al., 2005; Merner et al., 2008; Otten et al., 2010; van der Zwaag et al., 2012).

In addition, a variety of mutations in several ion channels that add to action potential generation have been described to trigger pro-arrhythmic remodeling of the heart. This subset of arrhythmogenic cardiac diseases is, however, beyond the scope of this review.

## Tissue architecture

Several extrinsic and intrinsic factors can lead to alterations in cardiac workload. Those changes can trigger growth of individual myocytes leading to cardiac hypertrophy. An increased amount of hypertrophy is associated with a decreased conduction velocity of the electrical impulse (Winterton et al., 1994; Cooklin et al., 1997; McIntyre and Fry, 1997). Moreover, metaplasia of fibroblasts into myofibroblasts, a more contractile and collagen producing cell type can increase the deposition of the extracellular matrix (ECM) leading to fibrosis (Davis and Molketin, 2013). This is further supported by an increased rate of cell death—necrosis and apoptosis—and inflammatory processes such as the secretion of TNF-alpha or IL-6 (Gill et al., 2002; Nian et al., 2004). Besides that, also alterations in myocardial fiber orientation can importantly affect characteristics of cardiac impulse propagation (Vetter et al., 2005).

### Hypertrophy and cell size

The key feature of hypertrophy in general is an increased cell size that electrically can be measured as an increase in cell-capacitance. Hypertrophy and increased cell size counteract the increased wall tension as caused by changes in cardiac workload (Laplace's law). Therefore, hypertrophy is seen as a compensatory mechanism. However, cardiac impulse propagation as well as conduction velocity gradually decrease with increasing severity of hypertrophy (Winterton et al., 1994; Cooklin et al., 1997; McIntyre and Fry, 1997) leading to an elevated risk for heart failure and sudden cardiac death (Meyerburg et al., 1992).

In DCM mainly the cell length is increased and this is associated by dilation and systolic disturbances of predominantly the left ventricle. Also both ventricles can be impaired, but generally with normal left ventricular wall thickness (Richardson et al., 1996; DuPont et al., 2001). The effect of eccentric hypertrophy on electrical signaling has been addressed in several animal models. An increase in conduction velocity was shown in a rabbit model, where a combined pressure- and volume overload increased heart weight by about 100%, and both cell length and width were increased by about 30%. Delayed activation was indicated by an increase in QRS duration from 50 ms in control to 58 ms in rabbits with DCM. Parallel and transverse to fiber orientation, however, epicardial conduction velocity appeared increased by 18 and 16%, respectively while transmural conduction velocity was unchanged. The authors of this study concluded that the increased cell size was responsible for the increase in longitudinal and transverse conduction velocity. Moreover, they concluded that the increased conduction velocity could not sufficiently compensate for the increased heart size, which was causative for the prolonged QRS durations (Wiegerinck et al., 2006). In a dog model of rapid pacing, it has been shown that QRS duration and cell length were increased, while cell width was reduced. Besides, transmural conduction velocity was reduced in both RV and LV, while Cx43 expression was reduced only in LV. As such, reduced cell width seems to play a dominant role in the reduced conduction velocity (Akar et al., 2004). In mice models, where DCM was induced by transverse aortic constriction (TAC), cell size, and the amount of hypertrophy was significantly increased after 6 weeks of TAC. This was leading to prolongations of PQ, QT, and QRS intervals, and slowing of right ventricular conduction velocity parallel to the fiber orientation (Boulaksil et al., 2010b). Data of a recent study with DCM patients showed elevated levels of myosin light-chain kinase and CRP that could possibly serve as diagnostic biomarkers for hypertrophy (Branishte et al., 2013).

In HCM, primarily cell width is increased (Formigli et al., 2003) leading to hypertrophy without dilation of the ventricles (Maron, 2002). This affects conduction of the electrical impulse as has been shown using computer modeling, where conduction velocity increased with cell size, and cell size was determined to be the dominant factor affecting conduction velocity (Spach et al., 2000). Also for HCM, several animal models have been described. In a rat model, where RV-HCM was induced by injections with monocrotaline (leading to pulmonary hypertension), increased cell width and lateralized Cx43 expression were found, while cell length was unaffected. Conduction velocity parallel to the fiber orientation was decreased, although perpendicular to the fiber orientation the conduction velocity was unchanged (Uzzaman et al., 2000). Similar results were obtained in another study that used the same rat model with monocrotaline induced pressure overload. In this study, RV cell width was increased, but again cell length remained unaffected. In the left ventricle, cell width and length were both decreased. Moreover, longer action potentials (at 90% repolarization), prolongation of the effective refractory period, and slowing of the longitudinal conduction velocity occurred (Hardziyenka et al., 2012). Besides the rat model, LV epicardial mapping was also performed in patients after chronic thromboembolic pulmonary hypertension. Comparable to the experimental model, these patients also showed prolongation of the effective refractory period and conduction slowing (Hardziyenka et al., 2012).

In ICM, cardiac hypertrophy can be found in the majority of patients (Kannel et al., 1961; Zaino and Tabor, 1963). In MRL (Murphy Roths Large) mice exhibiting an ICM phenotype, increased cell size and hypertrophy lead to a faster progression toward heart failure than in controls (Smiley et al., 2014). Moreover, electrocardiographic left ventricular hypertrophy embedded a predictive value for arrhythmias and mortality in ICM patients, making it an important factor to consider during diagnosis (Bender et al., 2012). In ACM no gross cardiac hypertrophy has been reported and the individual cell size is not increased either. Therefore, hypertrophy is not included as a parameter into the revised Task Force Criteria that are used to diagnose ACM (Marcus et al., 2010).

### Fibrosis and myocardial fiber disarray

In the healthy heart, cardiac myocytes are embedded in the ECM, a network of multiple molecules, proteins and thin intertwining strands of collagen fibers, synthesized by cardiac fibroblasts (Manabe et al., 2002). This network ensures tissue strength and allows cell-cell contact between neighboring cells (Weber et al., 1994). Cardiac fibrosis is the inappropriately high amount of collagen deposition in the heart during pathophysiological remodeling which hampers electrical conduction and enables the development of arrhythmias (Krenning et al., 2010). In addition, also cardiomyocyte fiber orientation plays an important role in the propagation of the electrical signal and fiber disarray can further facilitate the generation of ventricular arrhythmias (Brugada et al., 1991; Punske et al., 2005) This was recently shown in a study that combined data from neonatal rat cardiomyocytes and computer models (Kudryashova et al., 2014).

**Figure 1:**
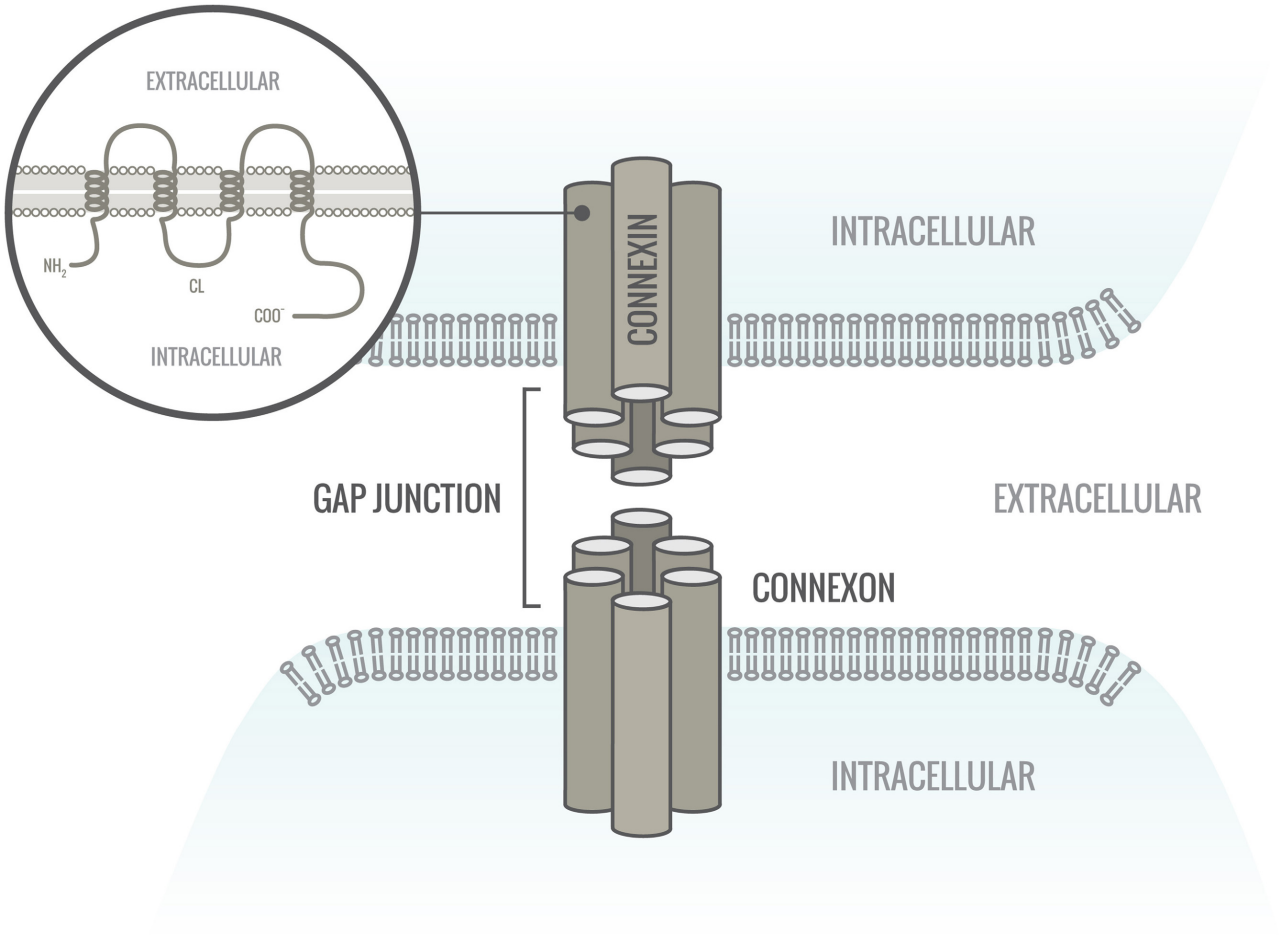
**The cardiac gap junction**. Gap junctions are located in the intercalated disc. They consist of two hemichannels (connexons) connecting across the intercellular space. Each hemichannel is provided by one cell and connexons are composed of six hexagonally arranged connexin proteins.

Fibrosis can be divided into replacement fibrosis (compact and patchy) and reactive fibrosis (interstitial and diffuse) (Swynghedauw, 1999). Typical examples of these four different forms we published before and are expressed in Figure 1 of a review article by the de Jong et al. (2011). Replacement fibrosis occurs after clearance of dead myocytes, where cardiac cells will be replaced by collagen fibers facilitating the preservation of the structure of the myocardium as well as the clearance of debris (de Jong et al., 2011). This normally includes compact or patchy fibrosis that is generated after an infarct or in due to chronic pressure overload. These processes have been described both in patients and in experimental animal models (Xia et al., 2009). Compact fibrosis is created, when a whole area is replaced by fibrosis and no viable myocytes are left. Although this seems to be dramatic for the cardiac contractile performance, this form is the least arrhythmogenic (de Bakker et al., 1988). Patchy fibrosis describes areas, where fibrosis and myocardial cells are confounded. In this case, collagen fibers are long strands disturbing the electrical signal propagation (de Bakker et al., 1993). Reactive fibrosis describes the process when more collagen is produced than degraded without a loss of viable cardiomyocytes. This includes interstitial and diffuse fibrosis and can be caused by mutations and changes in gene expression, a phenotypical switch from fibroblasts into myofibroblasts, as well as due to aging (Biernacka and Frangogiannis, 2011). Interstitial fibrosis is localized in between the individual cells. High interstitial collagen content causes reduced compliance and electrical impairment (Rohr, 2009) possibly leading to arrhythmias and heart failure (Janicki and Brower, 2002). Diffuse fibrosis is comparable to patchy fibrosis, however, the collagen strands are short and this form is less arrhythmogenic (Kawara et al., 2001).

In murine animal models that can be followed and analyzed at different time points after the experimental intervention has been made, chronic pressure overload initially leads to reactive fibrosis and in later stages this may change into a heterogeneous deposition of replacement fibrosis. The switch is likely caused by the fact that at a certain time point proper nutrient supply to the cells fails to maintain a minimal level (Isoyama and Nitta-Komatsubara, 2002).

Myocardial fiber disarray is the result of altered fiber orientation after e.g., an infarct. Adjacent cardiomyocytes are then mostly aligned in a perpendicular way or obliquely to each other in or around the collagen (Hughes, 2004). Nowadays, predominantly computer models are used to simulate possible alterations and effects on electrical propagation caused by fiber disarray. Computer models are by definition artificial and similarly to genetic engineered animal models, care should be taken by extrapolating these results to the human heart. However, these mathematical models may provide valuable additive insight, since they allow studying the effects induced by individual alterations but also the summation of more than one altered factor can be studied systemically. With such models models, risk of cardiac death can be predicted in e.g., long QT patients and various factors can be implemented at the same time like cell size, wall thickness, action potential duration and fibrosis (Hoefen et al., 2012; Zhao et al., 2013).

In DCM predominantly patchy areas of interstitial and replacement fibrosis can be found, but also perivascular patterns have been described (Nakayama et al., 1987; Swynghedauw, 1999). In HCM patients, interstitial fibrosis predominates (Swynghedauw, 1999). In mice with chronic pressure overload, next to hypertrophy, increased levels of interstitial fibrosis have been reported in many cases (Xia et al., 2009; Boulaksil et al., 2010a,b). Moreover, myocardial disarray is one of the hallmarks of HCM (Hughes, 2004). Although it is also present in other cardiac diseases and even in physiologically aged hearts, through the presence in high quantities it is specific for HCM making it a highly sensitive and useful marker (Hughes and McKenna, 2005).

In ICM, infarcted myocytes are replaced predominantly by heterogeneously distributed replacement- and interstitial fibrosis creating islands of cardiomyocytes surrounded by scar tissue. This so-called “labyrinth” of viable strands of cardiomyocytes surrounded by insulating areas of fibrosis creates disconnection of myocytes that leads to excitation blocks and arrhythmias (Fenoglio et al., 1983; Weber et al., 2008). In murine infarct models, fiber disarray has been reported in the infarct border zone, although to a much lesser extent than in HCM (Smith et al., 1991).

ACM is characterized by degeneration of cardiomyocytes and fibro-fatty replacement (Thiene et al., 1988). One theory is that altered cell-cell adhesion due to mutations in genes encoding for structures of the intercalated disc, e.g., the desmosomes, causes injuries to myocytes. This promotes cell death and leads to replacement by fibro-fatty tissue (Saffitz et al., 2009). Histologically, analysis of fibrosis and fiber orientation can help to diagnose any of the above described diseases. Moreover, in all diseases, fibrosis leads to conduction blocks, which increases the propensity to develop reentry arrhythmias, or promotes the manifestation of ectopic impulse generation in relatively uncoupled clusters of cardiomyocytes due to disease-induced remodeling of the expressed ion channels.

## Electrical coupling—gap junctions

Gap junctions in the heart are agglomerates of channels that connect the cytoplasm of two adjoining cells allowing electrical coupling between the cardiomyocytes as well as the exchange of certain small molecules, metabolites and ions up to a size of approximately 1 kDa (Elfgang et al., 1995; Noorman et al., 2009). One gap junction channel consists of two hemi-channels (connexons), each delivered by one of the two adjoining cells (Figure 1). The two connexons dock in the intercellular space to form a functional channel.

**Figure 2:**
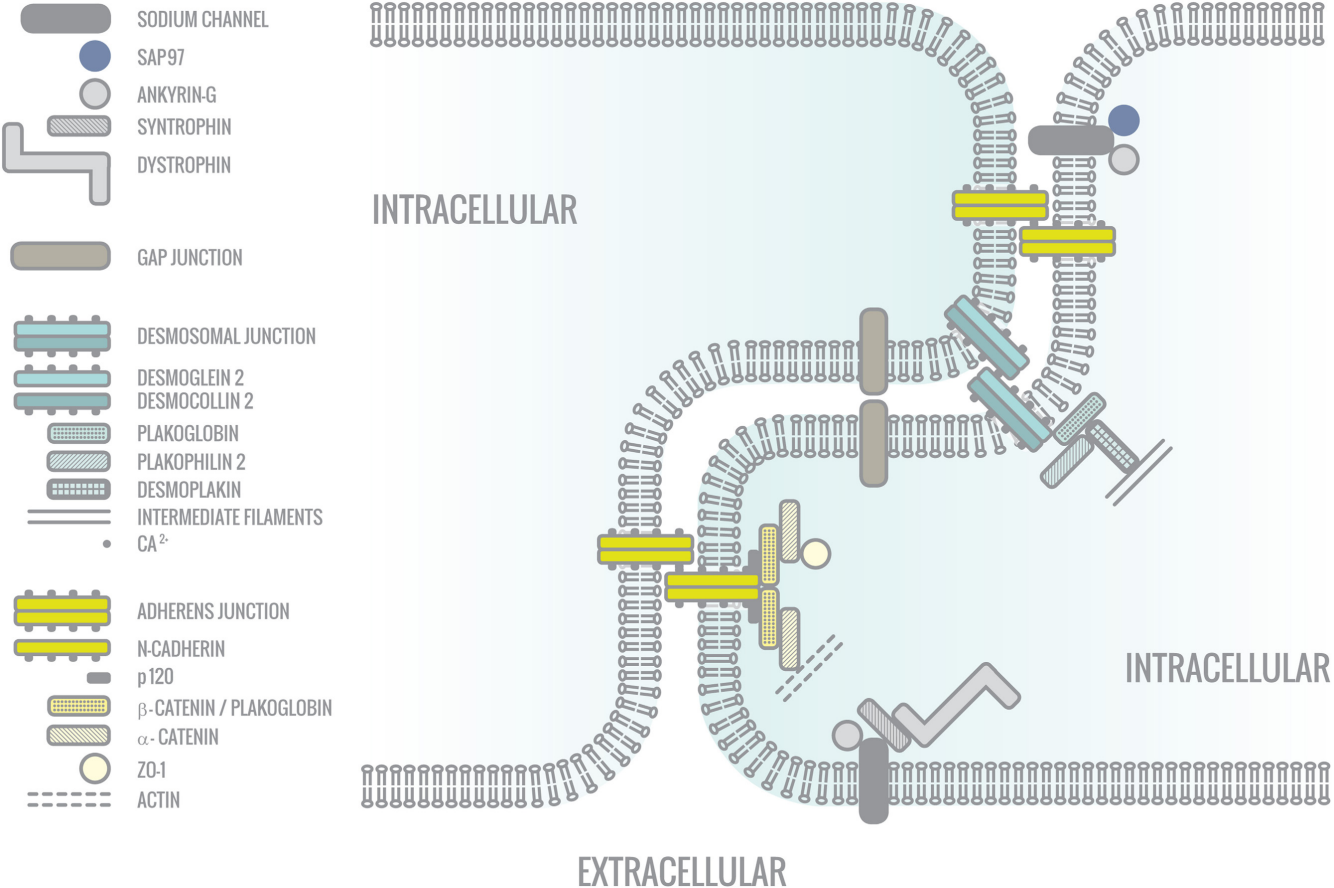
**The intercalated disc (ICD)**. The intercalated disc is the region between two cardiomyocytes, where different junctions are located: Gap junctions, adherens junctions, and desmosomal junctions as well as ion channels. They all form macromolecular protein complexes with different functions.

The connexons are composed of connexin proteins. In cardiomyocytes the three main isoforms that are expressed are Connexin 40 (Cx40), Connexin 45 (Cx45), and Connexin 43 (Cx43) with Cx43 being predominant in ventricular cardiomyocytes. Together with adherens junctions and desmosomes, gap junctions are localized in the intercalated disc (ICD), a step-like specialized membrane structure at the longitudinal cell edges between two cells (Figure 2). In those ICD's, gap junctions are normally present in the regions parallel (interplicate) to the longitudinal axis of the cardiomyocytes (Smith et al., 1991).

In patients of all four types of cardiac disease, a reduction in ventricular Cx43 localized at the ICD has been found (Peters et al., 1993; DuPont et al., 2001; Kaplan et al., 2004a). Using genetically engineered animal models, a plethora of data have been collected regarding to role of Cx43, using Cx43 knock out animal models. In 1995, the first Cx43 KO model revealed that these mice died perinatally due to major heart problems (malformation of the outflow tract) and cardiac failure (Reaume et al., 1995). Since then, conditional Cx43 KO mice and haploinsufficient animals have been generated. Nevertheless, results obtained from studies with heterozygous KO animals are contradicting. Mice showed reduced expression of Cx43 up to 50%, and in some studies this reduction resulted in reduced conduction velocity (Guerrero et al., 1997; Thomas et al., 1998; Eloff et al., 2001) while other studies in which a similar reduction in Cx43 was achieved no alterations in conduction velocity were reported (Morley et al., 1999; van Rijen et al., 2004; Stein et al., 2009, 2011). These contradicting results triggered to invent a different approach to reduce Cx43 expression, and also to circumvent the problem of perinatal death in homozygous KO mice. This was achieved through generation of two conditional KO mice models (Orban et al., 1992).

In the first model, one coding region of the Cx43 gene was replaced by the fusion construct Cre-ER(t) and the other Cx43 allele was flanked by loxP (Feil et al., 1996) resulting in a 50% reduced expression of Cx43 under basic conditions (Cx43^CreER(t)/fl^). Exposure to an agent activating the Cre recombinase (e.g., Tamoxifen) resulted in a further reduction of Cx43 expression up to maximally 95% (van Rijen et al., 2004). In the second model, the Cre gene was placed behind an alpha-myosin-heavy-chain (αMHC) promoter resulting in the deletion of the floxed Cx43 gene (αMHC-Cre/Cx43^fl/fl^) once the promoter got activated (Gutstein et al., 2001). In this way, the Cx43 gene was knocked out around birth allowing those animals to develop normally during gestational stages. In both models, mice died due to arrhythmias. In the case of the αMHC-Cre/Cx43^fl/fl^ mice death occurs around 1 or 2 months after birth (Gutstein et al., 2001) and in the case of the Cx43^CreER(t)/fl^ mice within 1 month after induction of the deletion (Eckardt et al., 2004).

In cell cultures of neonatal mouse cardiomyocytes with genetically reduced levels of Cx43, no differences in action potential amplitude or minimum diastolic potential could be found compared to wild type cells. However, dV/dtmax and action potential duration was increased (Thomas et al., 2003). Also intercellular conductance was reduced and propagation was slower and highly discontinuous (Beauchamp et al., 2004). In tissue strands composed of these cells and wild type cells, propagation velocity decreased significantly, when the amount of wild type cells was less than 50%. Again, excitation between wild the two types of cells was highly discontinuous (Beauchamp et al., 2012).

In humans, DCM is linked to a reduced expression of Cx43, especially seen together with lateralization (Kitamura et al., 2002, 2003; Kostin et al., 2003; Salameh et al., 2009). In animal models this observation was confirmed repeatedly. In mice with forced retinoic acid signaling (Hall et al., 2000), Cx43 expression was reduced and lateralized, with in one study some upregulation of Cx40 in the ventricles (van Veen et al., 2002). This led to an increase in QRS duration, a reduction in conduction velocity and an increased spatial dispersion of conduction velocity (Hall et al., 2000; van Veen et al., 2002). Cx43 downregulation was also confirmed in mice with a knock-out of muscle LIM protein (an acronym of the three gene products Lin-11, Isl-1 and Mec-3) (Ehler et al., 2001), and in a guinea pig model of chronic pressure overload (Wang et al., 1999). However, in another mouse model with longstanding pressure overload, no reductions in total Cx43 could be observed, but 44% of the animals displayed arrhythmias (Boulaksil et al., 2010a). In a rabbit DCM model of a combined volume and pressure overload, reduced mid-myocardial Cx43 was reported, QRS duration was prolonged and arrhythmias were inducible (Wiegerinck et al., 2006). Besides this animal model, another DCM rabbit model induced by volume overload showed a reduction in Cx43 expression in different groups (Goldfine et al., 1999; Haugan et al., 2006). In a dog model of DCM (induced by RV pacing), lateralized Cx43 expression was described and immunofluorescent signals were decreased in the left ventricle, but not in the right. Moreover, arrhythmias could be induced and QRS duration was prolonged. This was associated with a reduced longitudinal and transversal conduction velocity in both LV and RV (Akar et al., 2007). It was also reported that downregulation of Cx43 triggered fibrosis formation after pressure overload thereby connecting the different passive changes in the heart (Jansen et al., 2012a). The latter study showed that the severity of fibrosis could directly be related to the amount of Cx43 downregulation.

In HCM patients, in an early stage of the disease, an initial increase in Cx43 expression was described together with extensive lateral staining. In later stages, Cx43 was reduced and heterogeneously distributed (Kostin et al., 2004). Other groups showed a reduction in Cx43 without lateralization, or even lateralization without changes in Cx43 expression levels (Peters et al., 1993; Sepp et al., 1996). Animal models confirm some of the observed changes in humans. As already described in the hypertrophy section, in a rat model where HCM was induced using monocrotaline, a decrease in Cx43 expression at the ICD with lateralization was shown (Uzzaman et al., 2000) resulting in a, reduced longitudinal conduction velocity. The transverse conduction velocity and also cell width were unaltered. The reduced longitudinal conduction velocity was probably not caused by the overall diminishing of Cx43, because normally mild changes in coupling do not affect conduction velocity. Moreover, increased lateral expression of Cx43 and hypertrophy did not sufficiently lower resistance perpendicular to the fiber orientation to alter transversal conduction velocity (Palka et al., 1997; Jongsma and Wilders, 2000; Gutstein et al., 2001; van Rijen et al., 2004). More likely the altered source/sink ratios due to the changed axial/perpendicular resistance ratio might have influenced the conduction velocity. In another rat model, where chronically elevated pulmonary pressure triggered development of HCM, the overall amount of Cx43 remained the same, but it was heterogeneously distributed (Sasano et al., 2007). In two different rabbit models of HCM (one with a mutation in troponin 1 and one with the beta-MyHC-Q403 mutation), a significant increase in total mid-myocardial expression of Cx43 has been described, including phosphorylated Cx43 (Sanbe et al., 2005; Ripplinger et al., 2007). In an UM-X7.1 cardiomyopathic hamster model of HCM (leading to loss of cytoskeletal delta-sarcoglycan protein and therefore to cardiac remodeling, failure, and mortality) hypertrophy, a decrease in Cx43 mRNA, increased amounts of fibrosis and arrhythmias were seen after 20 weeks (Ambra et al., 2002; Sato et al., 2008). Cardiac hypertrophy and heart failure can also be induced by overexpression of constitutively active form of cardiac calcineurin-A (CnA) in the mouse heart. Next to hypertrophy these mice develop rapidly after birth extensive amounts of fibrosis and show a high incidence of arrhythmias (Molkentin et al., 1998). In this model reduced Cx43 signals in the ICD's and decreased conduction velocity can be observed (Bierhuizen et al., 2008). In mice undergoing TAC, heterogeneous and partly reduced signals of Cx43 have been reported leading to dispersed impulse conduction (Boulaksil et al., 2010a,b).

In ICM, ischemia reduces gap junction permeability (Dhein, 2006) and induces lateralization of Cx43 (Peters et al., 1993; Beardslee et al., 2000). The infarct border zone (the region bordering healthy and infarcted tissue) will, in that respect, be most at risk of remodeling. Already in 1991, Smith et al. reported gap junction remodeling in this zone, including reduced and lateralized Cx43 signals (Smith et al., 1991). This was also confirmed in dogs (Peters et al., 1997; Huang et al., 1999; Cabo et al., 2006), and patients (DuPont et al., 2001). In patients, alterations were even detected at areas distant from the border zone (Kostin et al., 2003). Moreover, an upregulation in Cx45 could be observed in the border zone, resulting in reduced gap junctional communication due to the intrinsic different properties of the Cx45 gap junction channels (Yamada et al., 2003). The observation of reduced and heterogeneously distributed Cx43 could also be confirmed in rabbit models (Tansey et al., 2006). However, in transgenic mice with 50% of the normal Cx43 expression level, the post-infarction area was smaller than in controls (Kanno et al., 2003). This would suggest that preservation of Cx43 is not *per se* beneficial for maintenance of function of the infarcted heart. Zhang et al. (2010) even proposed to be careful with increasing Cx43 expression in heart diseases until the meaning of alterations in expression of Cx43 upon myocardial infarction is fully understood. We have to keep in mind, however, that Cx43 gap junction channels not only are responsible for propagation of the electrical impulse in the heart, but also for metabolic coupling of the cardiomyocytes. In that respect, a smaller infarct size might rely on a reduced spreading of pro-apoptotic death signals due to a reduced level of intercellular coupling.

In ACM, reduced Cx43 expression has been reported in several studies. Theories are that Cx43 remodeling is triggered by abnormal mechanical coupling (Kaplan et al., 2004a) due to mutations in proteins composing junctions that support the structure of the cardiomyocytes. The different junctions in the intercalated disc assemble and form clusters. Therefore, dysfunction of one junction/protein influences the functioning of others (Agullo-Pascual et al., 2014). In human studies, heterogeneous disturbances in Cx43 signals, decreased presence in the intercalated disc, and lateralization of the signal has been reported in all different forms of ACM (Kaplan et al., 2004a; Noorman et al., 2013a). Although previously thought that this would predominantly be in the right ventricle, these changes are not exclusive and can also be found in the left ventricle and the septum (Noorman et al., 2013b). In general, this leads to scattered impulse conduction, which increases the susceptibility to arrhythmias (Boulaksil et al., 2010a). In the first attempts to investigate ACM in animal models, PKP2 mutations (the most abundant mutated desmosomal protein in humans) were used. In mice, homozygous null mutations in PKP2 are lethal whereas heterozygous mutations do not show any or only little phenotype (Ruiz et al., 1996; Grossman et al., 2004). Therefore, it is suggested that the abundant phenotypical presentation of ACM in patients is, on top of the genetic mutation, dependent on additional factors (e.g., inflammation, exercise). To study the familial cases, initially, two different familial forms of ACM have been studied with mutations in desmosomal genes different from PKP2: Naxos disease [mutation in plakoglobin (PKG)] (Protonotarios et al., 1986; McKoy et al., 2000; Protonotarios and Tsatsopoulou, 2004); and Carvajal-syndrome [desmoplakin (DPK)] (Carvajal-Huerta, 1998; Norgett et al., 2000). In patients, in both diseases, Cx43 is reduced in both ventricles in the early phase of the disease and an increase in non-phosphorylated Cx43 was detected that might be associated with accelerated down-regulation of the protein (Kaplan et al., 2004a,b). An *in vitro* model for Naxos disease using neonatal rat cardiomyocytes transfected with an adenovirus encoding the PKG 2057del2 mutation recapitulated all abnormalities seen in patients: reduced plakoglobin and Cx43 signal at the ICD's, increased apoptosis and secretion of inflammatory mediators (Asimaki et al., 2014). Moreover, a zebrafish model with the same mutation was used to screen for potential drugs to intervene with the development and progression of ACM (Asimaki et al., 2014). This latter study revealed promising results for future therapy since disease-causing targets could be identified and *in vitro* studies showed prevention and even regression of the disease when the intervention was applied in the early phases of cardiac remodeling.

## Excitability

Proper contraction of the heart also results from a finely tuned impulse generation, which feeds propagation. In ventricular cardiomyocytes, action potential generation is initiated by the fast and robust depolarization as caused by the opening of the voltage-dependent Na^+^ channels (Clancy and Kass, 2002). Already in 1921, Daly and Clark (1921) showed in frogs that rate, propagation velocity and force of the cardiac contraction were reduced in solutions containing a reduced (extra-cellular) sodium concentration.

**Figure 3:**
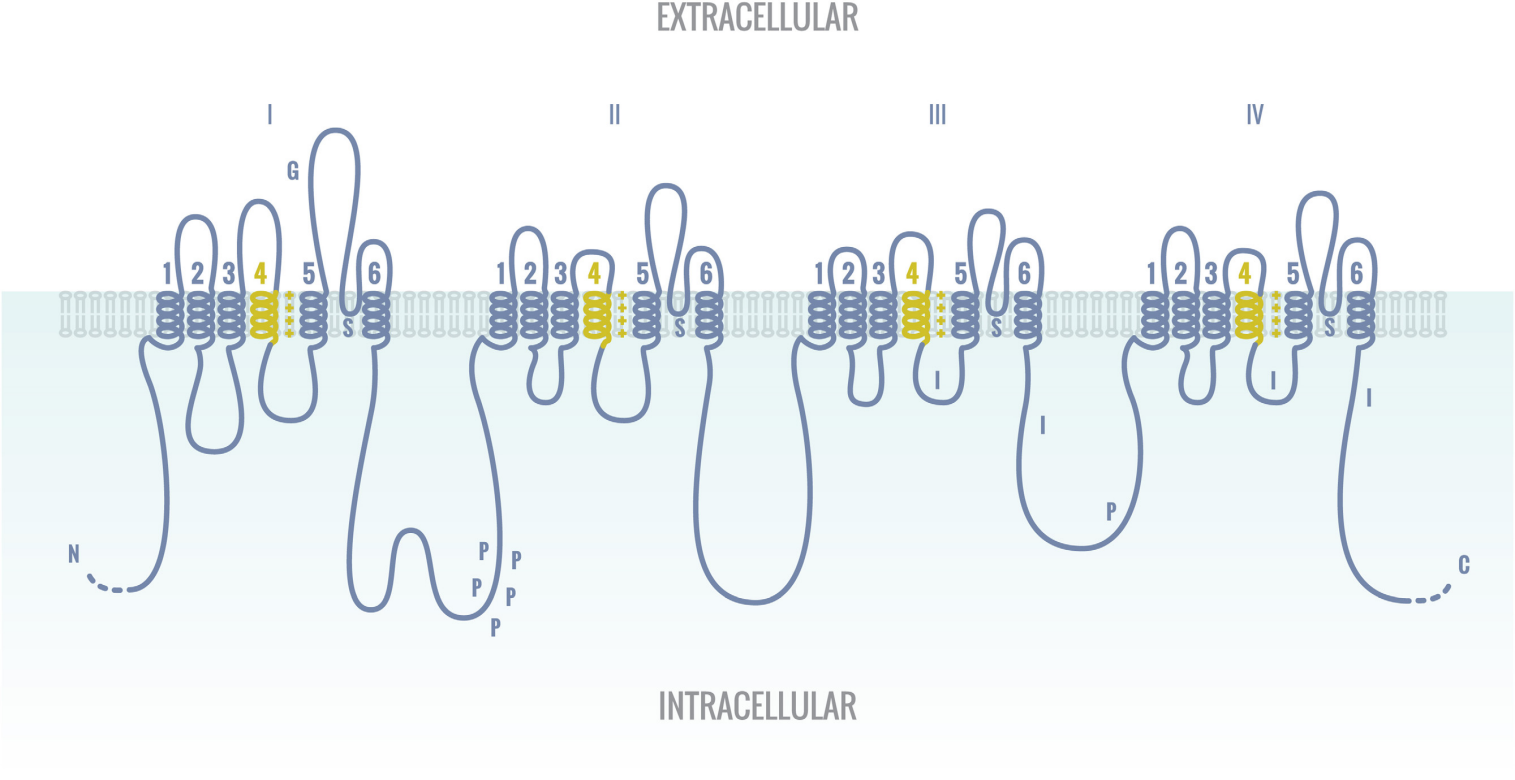
**The alpha subunit of the human sodium channel**. The alpha subunit of the human sodium channel consists of four homologous domains connected by cytoplasmic linkers. Each domain is composed of six trans-membrane sequences. G, glycosylation; P, phosphorylation; S, ion selectivity; I, inactivation sites. Positive (+) charges in S4 are crucial for transmembrane voltage sensing.

The human sodium channel consists of one alpha and four beta subunits. The alpha subunit consists of four homologous domains connected by cytoplasmic linkers (Figure 3). Each of those domains consists in turn of six transmembrane sequences (Balser, 1999). In cardiomyocytes two different pools of Nav1.5 channels co-exist, namely at the lateral membranes where the channel interacts with the dystrophin-syntrophin complex and in the intercalated disc where it binds SAP97 (Figure 2) (Petitprez et al., 2011). Both pools are thought to interact with different proteins. At the ICD, it is envisioned that Nav1.5 can influence e.g., Cx43 in the gap junctions and *vice versa* (Delmar, 2012).

Initially, much work on excitability has been performed *in vitro* using neonatal rat cardiomyocytes. Lateron, *in vivo* experiments on reduced excitability have been performed in genetically engineered mice models. SCN5a haploinsufficient mice showed about a 50% reduction of sodium current, while having a normal survival rate (Papadatos et al., 2002). ECG analysis showed a prolonged RR interval, P-wave duration, PR interval, QRS duration, and QT interval together with an age related aggravation of the phenotype (Royer et al., 2005). In those SCN5a haploinsuffient mice, QRS prolongation and conduction slowing was observed. However, in young mice, epicardial conduction velocity was not reduced in the left ventricle and only mildly reduced in the right. The reduction in conduction velocity became more dominant in both ventricles in older mice (12–17 months) where it was associated with increased fibrosis and altered Cx43 expression (van Veen et al., 2005).

In patients with the 1795InsD mutation in the SCN5a gene, bradycardia, conduction delay, QT prolongation, and right precordial ST-elevation can be detected. To investigate the cause of sodium channel related diseases, and in particular the above mentioned one, Remme et al. created a 1798InsD mouse model (1798InsD is the mouse analog of 1795InsD in human), where indeed a single mutation leads to a phenotype of bradycardia, right ventricular conduction slowing, and QT prolongation (Remme et al., 2006). Given the enormous amount of sodium channel mutations leading to a pro-arrhythmic phenotype in patients, generation of such mouse models significantly has contributed to a better understanding of the underlying etiology. In a DCM dog model, in which disease was induced through RV pacing, neither sodium current density, nor upstroke velocity of the action potential in left ventricular endo- or epicardial myocytes differed from control individuals (Kaab et al., 1996; Akar et al., 2004). Similarly, in a DCM rabbit model, sodium current density did not differ from controls in left ventricular myocytes (Wiegerinck et al., 2006). Recent data from a Torsade de Pointes sensitive canine heart model after chronic AV block (CAVB) confirm that no changes in I_Na_ appear, although increased cell size was reported (Boulaksil et al., 2011).

HCM patients showed increased CaMKII phosphorylation of Nav1.5 channels, which is in general associated with a delayed inactivation of the current (Wagner et al., 2006; Coppini et al., 2013). Increased late sodium current leads to prolonged repolarization (and therefore an increased action potential duration) and arrhythmias as has been shown in patients and several animal models (Jelicks and Siri, 1995; Undrovinas et al., 1999; Gray et al., 2001; Mészáros et al., 2001; Coppini et al., 2013). Increased late sodium current also causes changes in calcium metabolism and homeostasis, influencing cell metabolism and contractile functioning of the cells. This in turn may lead to hypertrophy (Coppini et al., 2013). In mice with calcineurin-induced cardiac hypertrophy, reduced Nav1.5 protein and RNA amounts could be found in the ICD's (Bierhuizen et al., 2008).

ICM is also associated with increased late I_Na_ (Ju et al., 1996; Huang et al., 2001). Various causes can lead to an increase of intracellular sodium concentration in ischemic cardiomyocytes. First, ischemia seems to increase the amplitude of the late I_Na_ significantly (Hammarstrom and Gage, 2002). Anaerobic glycolysis, due to lack of sufficient oxygen supply to the cells, leads to a decrease of ATP and leaking of protons via the cardiac Na/H exchanger (NHE1). Because of that, more sodium enters the cell (Tani and Neely, 1989). Moreover, a reduction of Na^+^/K^+^ ATPase pump activity will cause an additional decrease of sodium extrusion (Xiao and Allen, 1999). Increased sodium levels will activate the Na/Ca exchanger. This, in turn, leads to an increased concentration of calcium in the cell and a reduction in intracellular pH. The altered ion concentrations will lead to drastic changes in cellular metabolism, signal transduction, electrophysiological characteristics and contractile properties, probably leading to cell damage and cell death (Silverman and Stern, 1994; Imahashi et al., 1999; Allen and Xiao, 2002).

In the early stages of hypoxia, an increase in intracellular sodium concentration was observed in rat hearts that returned back to pre-infarction levels, when ischemia was of short duration (Tani and Neely, 1989). When sodium is persistently elevated, the subsequent increased calcium levels may be prevented by late sodium current inhibition through ranolazine (Soliman et al., 2012). Moreover, a NHE1 inhibitor has been reported to show cardio-protective effects after infarction in a rat model (Huber et al., 2012). Although controversial results have been published lateron about NHE1, it is still under investigation and thought to be one of the most effective post-infarct treatments (Karmazyn, 2013). Next to adherens-, gap- and desmosomal junctions, also ion channels are located in the ICD (Balse et al., 2012), and it becomes more and more clear that in ACM alterations in subcellular localization of sodium channels is importantly involved in the consequences of cardiac remodeling. In patients, a reduced localization of Nav1.5 in the ICD has been reported (Noorman et al., 2013a). In cultured cardiomyocytes, the reduced expression of PKP2, caused a subsequent reduction in the sodium current and a slower conduction velocity (Sato et al., 2009). Besides that, loss of expression of Ankyrin-G, an important protein that anchors the voltage-gated sodium channel, leads to disturbances in PKP2 and Cx43 expression. The other way around, loss of PKP2 causes a reduction in expression of Ankyrin-G as well as of Nav1.5 (Sato et al., 2011), thereby confirming the role of sodium channels in this disease. Besides that, in zebrafish and neonatal rat cardiomyocytes containing the mentioned 2057del2 plakoglobin (related to Naxos disease), a marked reduction in I_Na_ current density was observed (Asimaki et al., 2014). In these neonatal rat cardiomyocytes, the immunofluorescent Nav1.5 signal at the membrane was reduced, but total cell content appeared unchanged (Asimaki et al., 2014). This reduction in I_Na_ and consequently a reduced conduction velocity has also previously been shown in transgenic mice with a desmoglein-2 mutation (Rizzo et al., 2012). Moreover, reduced heterogeneous expression of Cx43 in conditional knockout mice caused decreased expression of Nav1.5, reduced sodium current and an increased vulnerability of those mice for arrhythmias (Jansen et al., 2012a). Similarly, PKP2 haploinsufficiency in mice resulted in a sodium current deficit and arrhythmogenesis when triggered with flecainide (Cerrone et al., 2012). The importance of these finding was reflected in a study of Cerrone et al. which showed that in patients with a mutation in PKP-2, provocation with flecainide triggered arrhythmias suggesting that also in these patients a concomitant sodium current deficit existed (Cerrone et al., 2014).

## Heterogeneity

The natural mode of functional heterogeneity within the normal cardiac performance is also of importance during heart disease. Due to, e.g., pulmonary disease or aortic stenosis, different parts of the heart are affected prior to the other, leading to heterogenic alterations that conflict with the natural organization. In ACM, where disease-dependent changes often start in the right ventricle, the left ventricle can remain relatively unaffected in the early stages of disease. In an opposite fashion aortic stenosis will first remodel the left ventricle before right ventricular involvement manifests. Differently, right ventricular remodeling due to pulmonary hypertension might simultaneously trigger LV remodeling (atrophy) due to a reduced filling (Hardziyenka et al., 2012).

These different types of remodeling are part of various diseases and are by definition heterogeneous, since all of those diseases have different causes and therefore treatment options (Coronel et al., 2013).

Cardiac remodeling deteriorating into heart failure most likely depends on a combination of the mechanisms as described in this article, and it is characterized by heterogeneous remodeling of excitation-contraction coupling (Coronel et al., 2001; Lou et al., 2011). Pathophysiological heterogeneity in cardiac tissue sustains ventricular tachycardia, stabilizes reentry arrhythmias and provides substrate for sustained tachycardia (Pazo et al., 2004; Ripplinger et al., 2006).

Passive heterogeneous ventricular remodeling in the heart is one of the most destructive features of cardiac remodeling and every remodeling mechanism itself shows the worst outcome, when occurring heterogeneously. In this article, several mechanisms of passive remodeling have been described during DCM, HCM, ICM and ACM. Passive remodeling of tissue architecture was investigated focusing on fibrosis, alterations in cell size, hypertrophy and fiber disarray. Disturbances in electrical coupling were discussed by describing changes in the distribution of the gap junction protein Cx43. Remodeling of electrical excitability was studied using the sodium channel protein Nav1.5.

To summarize, different types of fibrosis occur in all of the four discussed heart diseases and act as modulators of impulse propagation ranging from conduction slowing, increased dispersion of conduction and even conduction block. Moreover, it can alter the structure of the heart, e.g., alterations in cellular dimensions, which is caused by modifications in molecular pathways that control normal cellular physiology. As a consequence this may lead to reductions in excitability and cell-to-cell conduction (Fenoglio et al., 1983; Kawara et al., 2001). Heterogeneously distributed fibrosis can even create a “labyrinth” in the heart facilitating reentry arrhythmias (Engelman et al., 2010). Therefore, this is considered to be worse than only local interstitial fibrosis since it would be more difficult for electrical signals to propagate though a labyrinth of fibrotic patches than only being obstructed by single fibers (Kawara et al., 2001; Tanaka et al., 2007). This idea was strengthened by a study where reduction of heterogeneous patchy fibrosis was one-on-one correlated to a reduction of arrhythmias (Stein et al., 2010).

Hypertrophy can be detected in HCM and ICM. Changes in cell size are the key features of HCM and DCM, whereby HCM is characterized by an increase in cell width and DCM by an increase in cell length. Myocardial fiber disarray mainly occurs in HCM, where it is even used as a biomarker. It can also be observed in ICM, but to a much lesser extend. Heterogeneously distributed hypertrophy, myocardial fiber disarray and changes in cell size can lead to alterations in impulse propagation and conduction velocity causing disturbed signaling, deregulated contractions, and eventually heart failure (Meyerburg et al., 1992; Winterton et al., 1994; Cooklin et al., 1997; McIntyre and Fry, 1997). Pathophysiological heterogeneity in the sense of right vs. left ventricles or atria can also worsen disease phenotype as is classically illustrated by the fact that progressive LV dysfunction eventually results in RV failure too with consequently, pulmonary remodeling and edema (Chen et al., 2012).

Heterogeneous distribution, de-phosphorylation and a reduction of Cx43 signals in the ICD are characteristics found in all four different heart diseases. They are one of the key features of ACM (Noorman et al., 2013a), but also occur in DCM, ICM, and late stages of HCM. This can lead to a reduction in longitudinal and an increase in transverse conduction (reducing the normal anisotropic mode of conduction) causing heterogeneous propagation (increased dispersion of conduction) and arrhythmogenesis. At these places signaling (mechanical or electrical) will be more difficult leading to de-regulated intercellular coupling (Boulaksil et al., 2010a). The heterogeneous distribution of the different proteins and structures can be sub-divided into macro and micro heterogeneity. Macro heterogeneity compares the different regions in the heart, whereas micro heterogeneity is a measure for local heterogeneity (Jansen et al., 2012a).

Changes in expression and distribution of the sodium channel protein Nav1.5 can also be seen in HCM, DCM, and ACM. Homogeneous distribution of Nav1.5 throughout different parts of the heart is crucial for normal conduction (Clancy and Kass, 2002) although functional expression levels differ between epicard, endocard, and conduction system (Remme et al., 2009). Heterogeneous distribution, caused by a local increase or decrease of sodium channels possibly facilitates arrhythmogenesis and therefore heart failure, since the sodium current will be hampered or fastened locally (Wagner et al., 2011; Shryock et al., 2013). Although these heterogeneous maladaptations can have effects when occurring alone, they are often found to trigger arrhythmias and eventually heart failure due to a combination of simultaneous alterations in different factors. Therefore, great care should be taken when investigating one single mechanism of remodeling, since a heterogeneous adaptation of one mechanism can also cause others to develop or are even linked to each other. This concept is illustrated by several studies, which report a simulataneous downregulation and de-phosphorylation of Cx43, and Nav1.5, (Stein et al., 2009; Jansen et al., 2012b; Noorman et al., 2013a) and even link this combination to an increased deposition of fibrotic materials (van Veen et al., 2005; Jansen et al., 2012a). These studies example the complexity of cardiac remodeling during pathophysiology in relation to the increase in propensity to develop arrhythmias.

Regarding the future perspective of cardiac research, one should focus on these connections between heterogeneous changes in single factors within the heart that lead to the total picture of heterogeneous remodeling. This might unravel currently unknown relationships between the various forms of maladaptation and help to improve the search for treatment options. In a study that was mentioned before, long term administration of the antihypertensive drugs eplerenone or losartan in a mouse model of extreme aging not only reduced the amount of interstitial- and patchy fibrosis, but also preserved a homogeneous pattern of gap junctional coupling. This preservation of the normal substrate for conduction resulted in a significantly reduced amount of arrhythmias (Stein et al., 2010). Beyond pharmacological interventions, pacing strategies (like cardiac resynchronization therapy) that aim to correct the abnormal sequence of activation, e.g., due to bundle branch block or electrical remodeling, will provide further future perspectives to improve performance of the remodeled heart.

### Conflict of interest statement

The authors declare that the research was conducted in the absence of any commercial or financial relationships that could be construed as a potential conflict of interest.
